# Antimicrobial resistance and molecular genotyping of *Escherichia coli* and *Staphylococcus aureus* isolated from some Egyptian cheeses

**DOI:** 10.5455/javar.2021.h509

**Published:** 2021-06-19

**Authors:** Nahed Gomaa Kasem, Maha Al-Ashmawy, Mohammed Elsherbini, Adel Abdelkhalek

**Affiliations:** Department of Food Control and Hygiene, Faculty of Veterinary Medicine, Mansoura University, Mansoura, Egypt

**Keywords:** Antimicrobial resistance, E. coli, S. aureus, PCR, MAR index

## Abstract

**Objective:**

This work investigated the antimicrobial resistance (AMR) and virulence of *Escherichia coli* and *Staphylococcus aureus *in communally consumed cheeses in Egypt.

**Materials and Methods:**

This study examined 100 samples of Domiati, Tallaga, Cheddar, and Ras cheese collected from several shops and supermarkets. Samples were spread on selective media to isolate bacterial strains. Molecular characterization of bacterial isolates was carried out using polymerase chain reaction to determine Shiga toxin 1 (*stx1*), Shiga toxin 2 (*stx2*), *eaeA*, and* nuc *genes. The isolates were tested for susceptibility to 14 antibiotics by disk diffusion assay.

**Results:**

In this study, several *E. coli* serotypes were identified. *E. coli *O26:H11, O103:H2, and O111:H2 expressed *stx1/2*, *E. coli *O114:H4 expressed *stx1*, *E. coli *O17:H18, O21:H7 and O146:H21 expressed *stx2*, while only *E. coli* O26:H11 and O111:H2 expressed *eaeA*. The *E. coli* isolates were resistant to at least one antibiotic, while most isolates (82.4%) showed multidrug resistance (MDR). AMR to erythromycin was the highest (100%), followed by nalidixic acid (94.1%), cefotaxime (82.4%), vancomycin and cephalothin (64.7%), penicillin G (52.9%), sulfamethoxazole (47.1%), amikacin and kanamycin (35.3%), ampicillin (29.4%), tetracycline and ciprofloxacin (23.5%), and doxycycline (11.8%), while gentamicin showed the least resistance (5.9%). The multiple antibiotic resistance (MAR) index of the isolated *E. coli* ranged from 0.071 to 1 (mean = 0.478). All *S. aureus* isolates expressed the *nuc* gene and demonstrated resistance to at least one antibiotic, and 90% of isolates were MDR. AMR to kanamycin and cephalothin was the highest (100%), followed by penicillin (90%), doxycycline (70%), nalidixic acid and sulfamethoxazole (60%), erythromycin (50%), tetracycline, cefotaxime, and gentamicin (40%), ciprofloxacin and ampicillin (30%), and amikacin (20%). In comparison, vancomycin showed the least resistance (10%). MAR index of isolated *S. aureus *ranged from 0.143 to 1 (mean = 0.529).

**Conclusion:**

The antimicrobial-resistant *E. coli* and *S. aureus* are potential risks for public health and may have a role in disseminating AMR to other pathogenic and non-pathogenic microbes.

## Introduction

Currently, antimicrobial resistance (AMR) is a challenge that faces public health. It negatively influences the treatment of bacterial infections, resulting in increased death rates, morbidities, treatment costs, and decreased animals’ productivity [1]. In 2020 and beyond, AMR cannot be overlooked. At the global level, bacterial infections which are not effectively managed as a result of AMR influence approximately 700,000 individuals every year and probably result in 10 million deaths over 30 years, at the cost of US$100 trillion [2].

AMR, the silent worldwide pandemic, can worsen the coronavirus disease 19 (COVID-19) pandemic, which can exacerbate the AMR [2]. Data from five countries advocated that 6.9% of COVID-19-infected individuals had infections caused by bacteria (3.5% associated and 14.3% post-COVID-19) [3].

The antibiotics can be misused by healthcare personnel and the population resulting in a quick spreading of bacterial strains that resist antibiotics. Most bacterial strains that frequently result in infections in humans and animals have a high resistance degree to the first-line antibiotics [1].

The term AMR means the absence of response to a standard dose of an antibiotic. Bacterial strains show resistance to the antagonistic properties of antibiotic agents. They had former sensitivity, causing bacterial strains to survive despite using a standard dose of a particular antibiotic [4].

Multiple antibiotic resistance (MAR) index is an effective and cost-effective method for source tracking of bacterial strains having AMR. MAR index is the ratio between antibiotics’ number that a bacterial strain shows resistance to and the total antibiotics’ number the bacterial strain is exposed to. An MAR index exceeding 0.2 indicates an increased risk source of contamination where antibiotic agents are frequently used [5].

Milk and dairy products are rich sources of nutrients for humans worldwide. Various cheese types are formed worldwide. Cheeses are significantly consumed due to their high nutritional value [6]. The cheese quality is affected by equipment and environmental hygienic measures during manufacturing, packaging, and handling [7]. During cheese production, particularly during ripening, cheeses are exposed to unsterile environmental conditions where many opportunistic organisms, such as *Staphylococci*, *Escherichia coli*, and others, are reported [8].

Foodborne illness that might be associated with consuming cheese was reported in several regions worldwide. For instance, Honish et al. [9] concluded that *E. coli* caused a cheese-associated outbreak among 13 persons in Canada, resulting in two cases of hemolytic uremic syndrome. Additionally, and according to Delbes et al. [10], *Staphylococcus aureus* infection has been associated with the utilization of unpasteurized milk or with contamination related to unhygienic handling since these bacteria, when exceeding 5 Log colony-forming unit ml^−1^, release heat-resistant enterotoxin.

Studies carried out in the last 10 years revealed both the likelihood of AMR transmission via food chains and the significance of the food-handling environment as a possible hot spot for AMR development and dissemination [11]. Thus, investigating AMR in humans and animals is significant for detecting altering resistance patterns, applying control measures on antimicrobials misuse, and avoiding the spread of multidrug-resistant pathogens [12]. This work was conducted to detect the occurrence and molecular identification of *E. coli* and *S. aureus* in some Egyptian cheeses and determine the AMR of the bacterial isolates.

## Material and Methods

### Sample collection

The current work included 100 samples of Domiati, Tallaga, Cheddar, and Ras cheese (25 each) collected from different supermarkets between July 2019 and May 2020 in Egypt. All the samples were stored in pre-sterilized aseptic plastic containers with caps and were preserved in an ice-box at 4°C till they reached the laboratory.

### Isolation of E. coli and S. aureus

Based on the methodology described by Soomro et al. [13], *E.* c*oli* were isolated. In brief, 25 g from every sample was mixed with 225 ml of buffered peptone water, and homogenization was carried out for 3 min. Then, 0.1 m1 of the suitable dilutions of each sample was distributed onto MacConkey Agar plates (Oxoid, CM 0115) and incubated at 37°C for 24 h. Then, each plate was examined for the presence of viable *E. coli*. Five typical suspected colonies (round pink) were picked up for streaking onto MacConkey Agar. Incubation was carried out at 37°C for 24 h. To identify the *E. coli*, Gram-stain followed by microscopic examination and biochemical tests (indole, methyl-red, Voges–Proskauer, and citrate utilization) were carried out.

For the isolation of *S. aureus*, 0.1 ml of prepared dilutions of each sample was spread onto Baird–Parker plate and then distributed by surface plating method till complete absorption [14]. The plates were incubated at 37°C for 1–2 days and evaluated for *S. aureus* colonies.

### Serological identification of E. coli serotypes

Serotyping of *E. coli* was carried out using *E. coli* antisera sets (DENKA SEIKEN Co., Tokyo, Japan) [15].

### Bacterial deoxyribonucleic acid (DNA) extraction

DNA samples were extracted from the isolated bacteria using Fermentas GeneJET genomic DNA purification kit (Thermo Scientific, Australia), as stated by the manufacturer. DNA was preserved at −20°C till polymerase chain reaction (PCR) assay was carried out.

### Primers and multiplex PCR

The multiplex PCR was utilized to determine Shiga toxin 1 (*stx1), *Shiga toxin 2 (*stx2), and eaeA *in 17 *E. coli* isolates using the primers (Pharmacia Biotech) mentioned in Table 1. The procedure was carried out according to Paton and Paton [16]. A thermal cycler (Hamburg, Germany) was used to amplify 20 ng of DNA, and amplification was carried out in 25 ul of buffer solution, which contained 3 μM of oligonucleotides, 200 μM of each deoxynucleoside triphosphate (dNTP), 3.5 mM magnesium chloride, and 2.5 U of DNA *Taq *polymerase. A total of 35 cycles of PCR were carried out. In every cycle (for the initial 10 cycles), DNA was denatured at 95°C for 1 min, annealed at 65°C for 2 min, decremented to 60°C at cycle number 15, elongated at 72°C for 90 sec, and incremented for 2.5 min from cycle 25 to cycle 35. The entire PCR amplification products were separated on 1.5% agarose gel and were stained using ethidium bromide to visualize using an ultraviolet light transilluminator.

**Table 1.** Primers utilized to identify *E. coli *genes.

Gene	Primer (5′→3′)	Size	References
***S****tx1***-F**	5′-ATAAATCGCCATTCGTTGACTAC-3′	180 bp	[15]
***Stx1*-R**	5′-AGAACGCCCACTGAGATCATC-3′		
***Stx2*-F**	5′-GGCACTGTCTGAAACTGCTCC-3′	255 bp	
***Stx2*-R**	5′-TCGCCAGTTATCTGACATTCTG-3′		
***eaeA-F***	5′-GACCCGGCACAAGCATAAGC-3′	384 bp	
***eaeA-R***	5′-CCACCTGCAGCAACAAGAGG-3′		

The primers of *nuc *utilized for the detection and identification of *S. aureus* are shown in Table 2. The procedure was carried out according to the method described by Chu et al. [17]. The amplification was carried out on the thermal cycler utilizing 25 μl of PCR mix that contained 3 μl of boiled cell lysate, 200 μM of dNTP, 1.4 U of *Taq* DNA polymerase (Biotools, Spain), buffer (20 mM Tris-hydrochloride pH 8.4, 50 mM potassium chloride and 3 mM magnesium chloride), and 20 μM of each primer (*nuc*). The amplification program included denaturation for 5 min at 94°C. Denaturation was carried out for 25 cycles at 94°C for 45 sec, followed by annealing at 55°C for another 45 sec, and eventually extension at 72°C for 10 min. 

### Antimicrobial susceptibility of E. coli and S. aureus

This was carried out using Mueller Hinton agar-based agar disk-diffusion testing. Various concentrations of sensitivity disks (Oxoid Limited, Basingstoke, Hampshire, United Kingdom) were used. Antibiotic classes comprised tetracycline (tetracycline, doxycycline), penicillin (ampicillin, penicillin G), macrolide (erythromycin), sulfonamide (sulfamethoxazole), cephalosporin (cefotaxime, cephalothin), aminoglycoside ( kanamycin, amikacin, and gentamicin), fluoroquinolones (nalidixic acid, ciprofloxacin), and glycopeptide (vancomycin) (Table 3). Inhibition zones on plates were measured depending on the Clinical and Laboratory Standard Institute’s guidelines [18]. Multiple drug resistance was reported as resistance to ≥3 antibiotics [19].

**Table 2.** Primers utilized to identify *S. aureus *gene.

*Gene*	Primer (5’→3’)	Size	References
***nuc-F***	5’-GCGATTGATGGTGATACGGTT-3’	270 bp	[37]
***nuc-R***	5’-AGCCAAGCCTTGACGAACTAAAGC-3’		

### Determination of MAR index

MAR index was calculated as follows: Number of antimicrobials showing resistance divided by the number of utilized antimicrobials [5].

## Results and Discussion

Cheeses are widely consumed dairy products in Egypt. It supplies protein, fat, vitamins, and minerals to the consumer. However, the cheese might be contaminated during its manufacture, distribution, and/or storage [20]. Due to their unique composition and properties, these may act as rich growth media for pathogens. *Staphylococcus aureus a*nd *E. coli *are the most commonly occurring potential microbes in the milk or dairy industry. They are thus the major bacteriological hazards associated with milk and cheese consumption [21].

The current work identified *E. coli* in 86.6% of Tallaga samples, 85.7% of Domiati samples, 52.1% of Cheddar samples, and 38.8% of Ras cheese samples (Table 4). Soft cheeses were highly contaminated with *E. coli* than hard cheeses (Ras cheese), which might be due to the high moisture of soft cheese than that of hard cheese and its shorter shelf-life due to bacterial spoilage. It was demonstrated that most soft and unripened cheeses are bacteriologically unstable because of the metabolic activities of bacterial strains [22]. It should be noted that according to Egyptian Standard (2005), cheese must be free from *E*. *coli* [23]. Accordingly, the four types of cheeses used in this study did not fulfill the Egyptian standards. Regarding the incidence of *S. aureus* in cheeses, 60% of Tallaga samples, 48% of Domiati samples, 48% cheddar samples, and 72% of Ras cheese samples were associated with *S. aureus* (Table 5).

**Table 3.** Antibiotic disks, concentrations, and action on pathogens.

Antibiotic	Sensitivity disc content (ug)	Resistant (mm)	Intermediate (mm)	Susceptible (mm)
**Amikacin**	30	≤12	13–15	≥16
**Penicillin G**	10 IU	≤20	21–28	≥29
**Gentamicin**	10	≤12	13–14	≥15
**Doxycycline**	30	≤14	15–18	≥19
**Kanamycin**	30	≤13	14–17	≥18
**Vancomycin**	15	≤15	16–21	≥22
**Nalidixic acid**	30	≤13	14–18	≥19
**Ciprofloxacin**	5	≤15	15–19	≥20
**Tetracycline**	30	≤14	15–18	≥19
**Erythromycin**	15	≤13	14–22	≥23
**Cefotaxime**	30	≤17	18–22	≥23
**Ampicillin**	10	≤13	14–17	≥18
**Cephalothin**	30	≤14	15–17	≥18
**Sulphamethoxazole**	25	≤10	11–15	≥16

**Table 4.** Incidence of *Enterobacteriaceae* and *E. coli *in cheese samples.

Cheese type	Total samples	Positive samples (%)	Total isolates	*E. coli* (%)	Enterobacter (%)	Shigella (%)	Yerisinia (%)	Klebsiella (%)	Proteus (%)
**Tal**l**aga**	25	12 (48%)	30	26 (86.6%)	2 (6.6 %)	1 (3.3%)	1 (3.3%)	–	–
**Domiat**i	25	8 (32%)	14	12 (85.7%)	2 (14.2 %)	–	–	–	–
**Chedd**a**r**	25	9 (36%)	23	12 (52.1%)	10 (43.4%)	1 (4.3%)	–	–	–
**Ras**	25	18 (72%)	54	21 (38.8%)	26 (48.1%)	3 (5.5%)	–	2 (3.7%)	2 (3.7%)

**Table 5.** Incidence of *S. aureus *in cheese samples.

Cheese type	Total samples	Positive samples (%)
**Tal**l**aga**	25	15 (60%)
**Domiat**i	25	12 (48%)
**Chedd**a**r**	25	12 (48%)
**Ras**	25	18 (72%)

**Table 6.** Serological characterization of *E. coli *isolates (*n* = 17).

Strain	No. (%) of isolates	Identified serotypes
**EPEC**	7 (41%)	O146:H21, O17:H18, O119:H6, O119:H6, O146:H21, O119:H6, O114:H4
**EHEC**	5 (29%)	O121:H7, O26:H11, O103:H2, O111:H2, O26:H11
**ETEC**	4 (24%)	O128:H2
**EIEC**	1 (6%)	O159

A notable difference in prevalence was found between the results of this study and previous reports. Differences in preparation procedures, storage, type of cheese, and whether milk was raw or pasteurized might be responsible for such discrepancies. In addition, this is probably because of the unhygienic measures taken where cheeses are produced and workers involved in the process [24]. Al-Gamal et al. [25] evaluated Tallaga cheese, Ras cheese, Domiati cheese, and Feta cheeses in Egypt and reported that 26.6% had *E. coli. *In Iran, among 77 soft cheese samples, *E. coli* could be isolated in 76 (98.70%) samples, of which 15 (19.48%) isolates were Enteropathogenic *E. coli* (EPEC) [26]. Ombarak et al. [27] isolated *E. coli* in 22% of Ras cheese. In Egypt, Younis et al. [28] isolated *E. coli* and* S. aureus* in 56%, 88%, 68%, and 76% of Tallaga and Ras cheeses samples, respectively. A study examined soft cheese samples in Brazil and reported that *S. aureus* was detected in 20% of samples, and EPEC was detected in about half of the total samples (49.1%) [29]. Abdel-Hameid Ahmed et al. [30] detected *S. aureus* in 14% of Domiati cheese. In Iranian research, authors detected *S. aureus* in 22.5% of 100 cheese samples [31]. Abulreesh and Organji [32] detected *S. aureus* in Ras cheese samples collected in Saudi Arabia.

In our study, 41% of the *E. coli* isolates were identified as EPEC (main pathotype), 29% as Enterohemorrhagic *E. coli* (EHEC), 24% as Enterotoxigenic *E. coli* (ETEC), and 6% as Enteroinvasive *E. coli* (EIEC) (Table 6). Consistent with our findings, a study in Iraq revealed that 40.5% of cheese samples showed contamination with EPEC [33]. 

EPEC strain was detected as O146: H21, O17: H18, O119: H6, and O114: H4. EHEC strain was detected as O26: H11, O111: H2, O103: H2, and O121: H7. ETEC strain was detected as O128: H2. EIEC strain was detected as O159. The results indicated that O128: H2 was the most prevalent serotype, followed by O119: H6 (Table 7). *E. coli* isolation is a major public health concern as some strains belong to enteropathogenic or toxigenic or both types [34].

The incidence of *E. coli *in cheeses might be related to fecal contamination or unhygienic measures in the cheese manufacturing process [35]. Many *E. coli *strains might result in gastrointestinal illness in humans. Among them are O157, O26, O103, O111, O145, O45, O55, O91, O113, O121, and O128 serotypes [36]. To overcome this problem, milk pasteurization is recommended during cheese production, as supported by the Egyptian Organization for Standardization and Quality Control.

**Figure 1.**
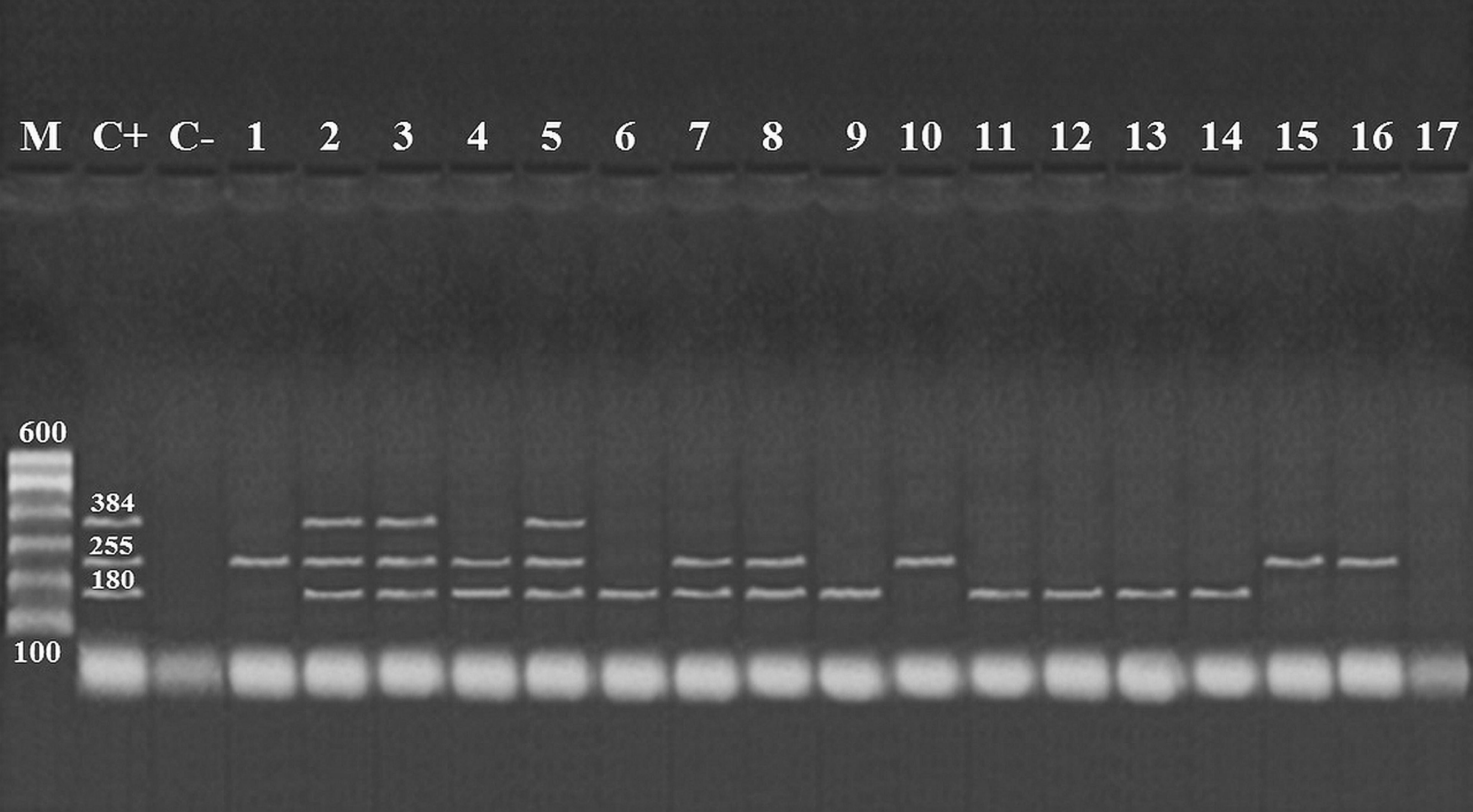
Multiplex PCR for *stx1* (180 bp), *stx2* (255 bp), and *eaeA* (384 bp) to identify *E. coli*. Lane-M: 100 bp DNA ladder; Lane-C+: positive control; Lane-C−: negative control; Lanes 2, 3 (*O26*), and 5 (*O111*): positive *E. coli* for *stx1*, *stx2*, and *eaeA* genes; Lanes 4 (O103), 7, and 8 (O119): positive *E. coli* for *stx1* and *stx2* genes; Lanes 6 (*O114*), 9 (*O119*), 11, 12, 13, and 14 (*O128*): positive *E. coli* for *stx1*; Lanes 1 (*O17*), 10 (*O121*), 15, and 16 (*O146*): positive strain for *stx2*; and Lane 17 (O159): negative *E. coli* for *stx1*, *stx2*, and *eaeA*.

The expression of *stx1*, *stx2*, and *eaeA* by *E. coli *was examined by the multiplex-PCR (Fig. 1). The results revealed that 12 (70.58%), 10 (58.82%), and 3 (17.6%) of *E. coli* isolates contain *stx1*,* stx2*, and* eaeA *singly, respectively (Table 5). Also, only 2 *E. coli* serovars that expressed *eaeA* gene were O26: H11, and O111: H2; both contained all the three virulence genes. On the other hand, serovars O17: H18, O121: H7, O146: H21, and O159 did not express *stx1* gene, while O114: H4, O128: H2, and O159 did not express *stx2* gene. By comparison, El-Badry and Raslan study [20] reported that O127:H6 strain expressed *stx1* and *stx2 *genes, whereas O111:H4 strain expressed *stx1* only O124: H and O55:H7 strains only expressed *stx2*. Besides, Fadel and Ismail [37] in Egypt detected several *E. coli* serovars in Ras and Kareish cheeses, which included O15:H11, O22:H5, O25: NM, O26:H11, O86:H34, O91:H10, O113:H21, O114:H2, O119:H6, O124:H7, O128:H2, O127: NM, and O145: NM. Moreover, El Bagoury et al. [38] isolated O26:H11, O111:H2, O124, O163:H2, O114, O125:H21, and O1, O15, along with a non-typed serotype in cheese samples (Tallaga, Karish, and Domiati).

**Table 7.** Occurrence of virulence genes of *E. coli *isolates (*n* = 17) in cheese samples.

Serotype	No. (%) of isolates	Stx1		Stx2		Intimin gene (*eaeA*)	
		No.	%	No.	%	No.	%
**O17: H18**	1 (5.8%)	0	0	1	100	0	0
**O26: H11**	2 (11.76%)	2	100	2	100	2	100
**O103: H2**	1 (5.8%)	1	100	1	100	0	0
**O111: H2**	1 (5.8%)	1	100	1	100	1	100
**O114: H4**	1 (5.8%)	1	100	0	0	0	0
**O119: H6**	3 (17.6%)	3	100	2	66.7	0	0
**O121: H7**	1 (5.8%)	0	0	1	100	0	0
**O128: H2**	4 (23.5%)	4	100	0	0	0	0
**O146: H21**	2 (11.7%)	0	0	2	100	0	0
**O159**	1 (5.8%)	0	0	0	0	0	0
**No. (%)**	17	12	70.58%	10	58.82%	3	17.6%

**Figure 2.**
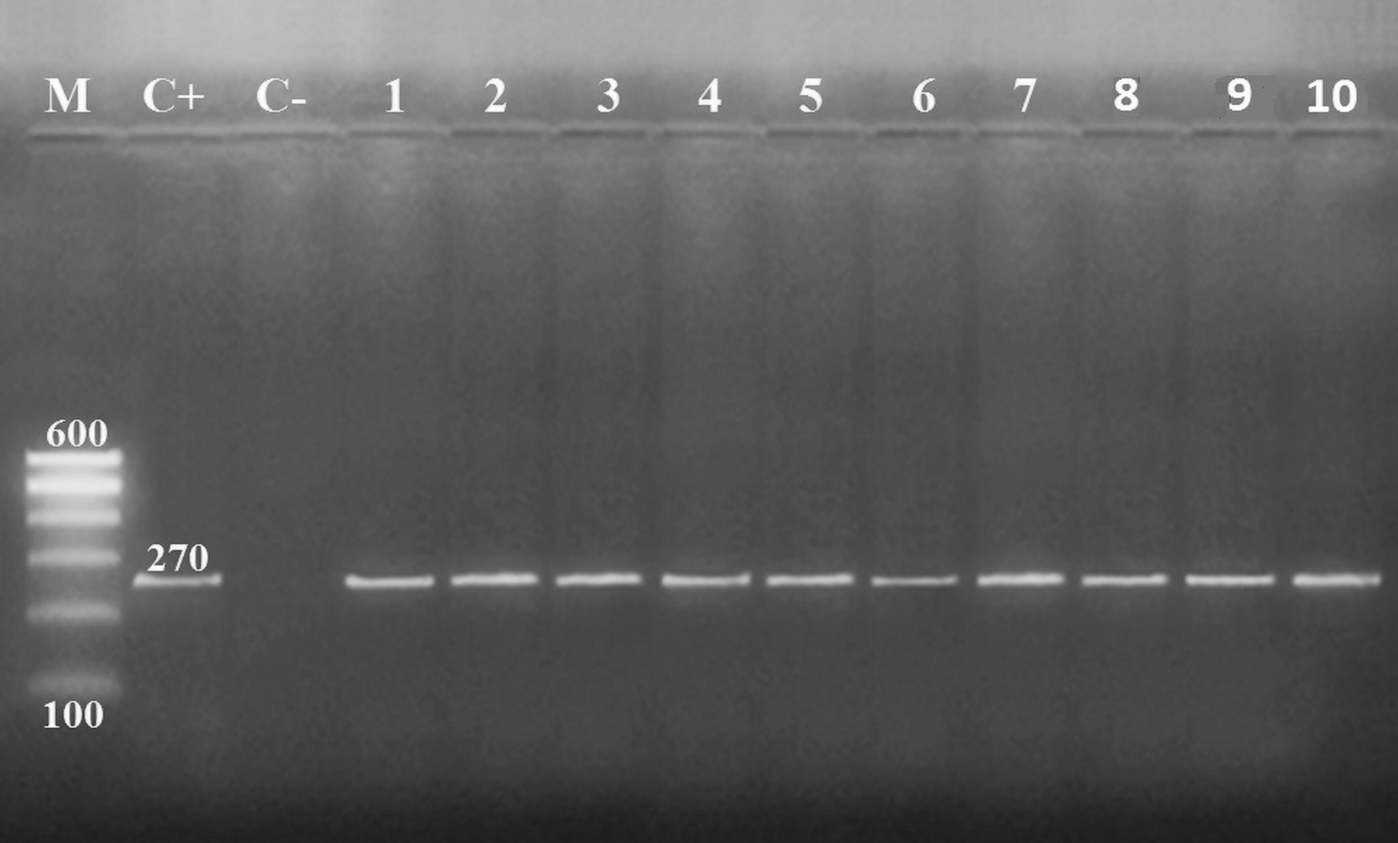
PCR of *nuc* (270 bp) aimed at *S. aureus *identification. Lane-M: 100 bp DNA ladder. Lane-1: positive control; Lane-2: negative control; Lanes 1–10: positive for *nuc* gene.

Regarding the molecular characterization of *S. aureus* in this study, PCR was used to recognize the *nuc* gene in *S. aureus* isolates (*n* = 10). As shown in Figure 2, all *S. aureus* isolates (100%) expressed the *nuc* gene. Considering the findings of Brakstad et al. [39], in comparison with our study, it can be stated that PCR for* nuc *gene amplification has the potential for quick diagnosis and confirmation of *S.aureus *isolates.

**Figure 3.**
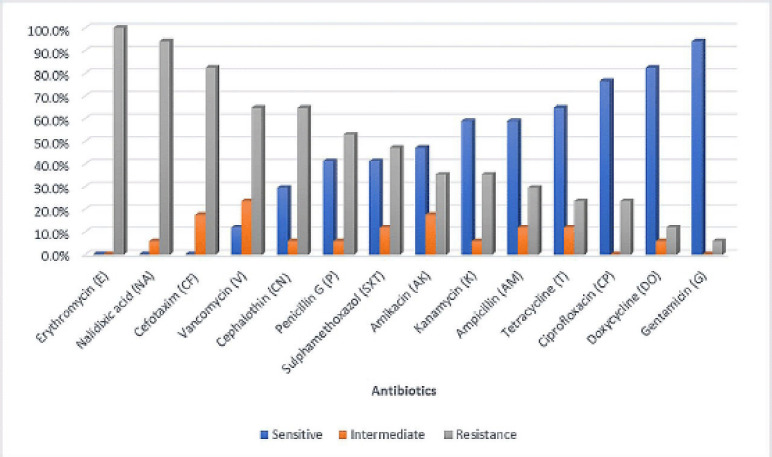
Antibiotic susceptibility of isolated *E. coli*.

The AMR patterns of *E. coli* are shown in Figure 3. All isolates had AMR to at least one antibiotic, while 82.4% of them showed multidrug resistance (MDR) (MAR index above 0.2) (Tables 8 and 9). Elafify et al. [40] found a near similar result, and reported that 86.11% of *E. coli* isolates in Egyptian cheeses were MDR. Other studies detected MDR* E. coli *with various ratios*. *For instance, in Egypt*, *Ombarak et al. [41] stated that half of the *E. coli* isolated from Karish and Ras cheeses were MDR. In Ethiopia, Bedasa et al. [42] recorded a higher MDR of *E. coli *isolates (92.5%) in comparison with our results. These differences among MDR *E. coli* might be associated with dissimilarities in antimicrobials used at the regional level.

Our study showed that AMR and MDR are prevalent in *E. coli* isolated from cheese samples. AMR to erythromycin was the highest (100%), followed by nalidixic acid (94.1%), cefotaxime (82.4%), vancomycin (64.7%), cephalothin (64.7%), penicillin G (52.9%), sulfamethoxazole (47.1%), amikacin (35.3%), kanamycin (35.3%), ampicillin (29.4%), tetracycline (23.5%), ciprofloxacin (23.5%), doxycycline (11.8%), and gentamicin (5.9%). The MAR index ranged from 0.071 to 1 (average 0.478). Compared to other techniques like genotypic characterization, the MAR index is cost-effective, quick, and reliable. Besides, it is simple and not necessitating specific skills or costly equipment [5]. The detection of resistant *E. coli* is critical since this can increase bacteria that can resist antibiotic drugs [43].

**Table 8.** Antibiotic resistance of *E. coli *isolates.

No.	Strain	Antibiotic resistance	MAR index
**1**	O128: H2	Erythromycin, Nalidixic acid, Cefotaxime, Vancomycin, Cephalothin, Penicillin, Sulphamethoxazole, Amikacin, Kanamycin, Ampicillin, Tetracycline, Ciprofloxacin, Doxycycline, Gentamicin	1
**2**	O128: H2	Erythromycin, Nalidixic acid, Cefotaxime, Vancomycin, Cephalothin, Penicillin, Sulphamethoxazole, Amikacin, Kanamycin, Ampicillin	0.714
**3**	O128: H2	Erythromycin, Nalidixic acid, Cefotaxime, Vancomycin, Cephalothin, Penicillin	0.428
**4**	O128: H2	Erythromycin, Nalidixic acid, Cefotaxime	0.214
**5**	O119: H6	Erythromycin, Nalidixic acid, Cefotaxime, Vancomycin, Cephalothin, Penicillin, Sulphamethoxazole, Amikacin, Kanamycin, Ampicillin, Tetracycline, Ciprofloxacin, Doxycycline	0.928
**6**	O119: H6	Erythromycin, Nalidixic acid, Cefotaxime, Vancomycin, Cephalothin, Penicillin, Sulphamethoxazole	0.500
**7**	O119: H6	Erythromycin, Nalidixic acid	0.134
**8**	O26: H11	Erythromycin, Nalidixic acid, Cefotaxime, Vancomycin, Cephalothin, Penicillin, Sulphamethoxazole, Amikacin, Kanamycin, Ampicillin, Tetracycline, Ciprofloxacin	0.857
**9**	O26 : H11	Erythromycin, Nalidixic acid, Cefotaxime, Vancomycin, Cephalothin	0.357
**10**	O146: H21	Erythromycin, Nalidixic acid, Cefotaxime, Vancomycin, Cephalothin, Penicillin, Sulphamethoxazole, Amikacin, Kanamycin, Ampicillin, Tetracycline, Ciprofloxacin	0.857
**11**	O146: H21	Erythromycin, Nalidixic acid, Cefotaxime	0.214
**12**	O111: H2	Erythromycin, Nalidixic acid, Cefotaxime, Vancomycin, Cephalothin, Penicillin, Sulphamethoxazole, Amikacin, Kanamycin	0.643
**13**	O17: H18	Erythromycin, Nalidixic acid, Cefotaxime, Vancomycin, Cephalothin, Penicillin, Sulphamethoxazole	0.500
**14**	O103: H2	Erythromycin, Nalidixic acid, Cefotaxime, Vancomycin, Cephalothin	0.357
**15**	O159	Erythromycin, Nalidixic acid, Cefotaxime	0.214
**16**	O121: H7	Erythromycin, Nalidixic acid	0.143
**17**	O114: H4	Erythromycin	0.071
Average = 0.478			

**Table 9.** Distribution of MDR of *E. coli* isolates (*n* = 17).

Item	Number	%
**Isolates with MDR (MAR index >0.2)**	14	82.4
**Isolates without MDR (MAR index < 0.2)**	3	17.6

In harmony with our findings, El Bagoury et al. [38] analyzed the antibiotic susceptibility of some isolated *E. coli*. They reported that *E. coli* is mainly resistant to erythromycin (100%), and it was most susceptible to gentamicin (77.8%). Sulfamethoxazole and oxytetracycline demonstrated intermediate susceptibility at percentages of 55.6% and 44.4%, respectively. Also, they revealed that most *E. coli* strains showing resistance were O26:H11, while *E. coli* O15 was resistant to erythromycin only.

On the contrary, Rahimi et al. [44] revealed *E. coli* resistant to ampicillin (44.4%), gentamycin (44.4%), erythromycin (33.3%), amoxicillin (11.1%), nalidixic acid (1.1%), and tetracycline (11.1%). Besides, Gundogan and Avci [45] found *E. coli* resistant to ampicillin (90.5%) and penicillin (82.1%). Also, they reported that the AMR was 58.4% for erythromycin, 53.7% for gentamicin, 44.2% for trimethoprim/sulfamethoxazole, and 29.4% for chloramphenicol.

The greatest MAR index for *E. coli* isolates was 1 (for O128: H2) (Table 6). This indicates the high resistance of *E. coli* bacteria in Egyptian cheeses. In most developing countries, like Egypt, the low cost and the wide availability of such antibiotic drugs are the primary reasons for their high utilization in treating diseases, predominantly diarrhea [46]. MDR strains can directly infect humans from the food chain or through animal contact or indirectly from environmental sources [47]. In recent times, there is a considerable increase in foodborne pathogens showing resistance to many antibiotics and as a result of extensive antibiotics’ usage in farming and through food chains which are known AMR sources [48].

**Figure 4.**
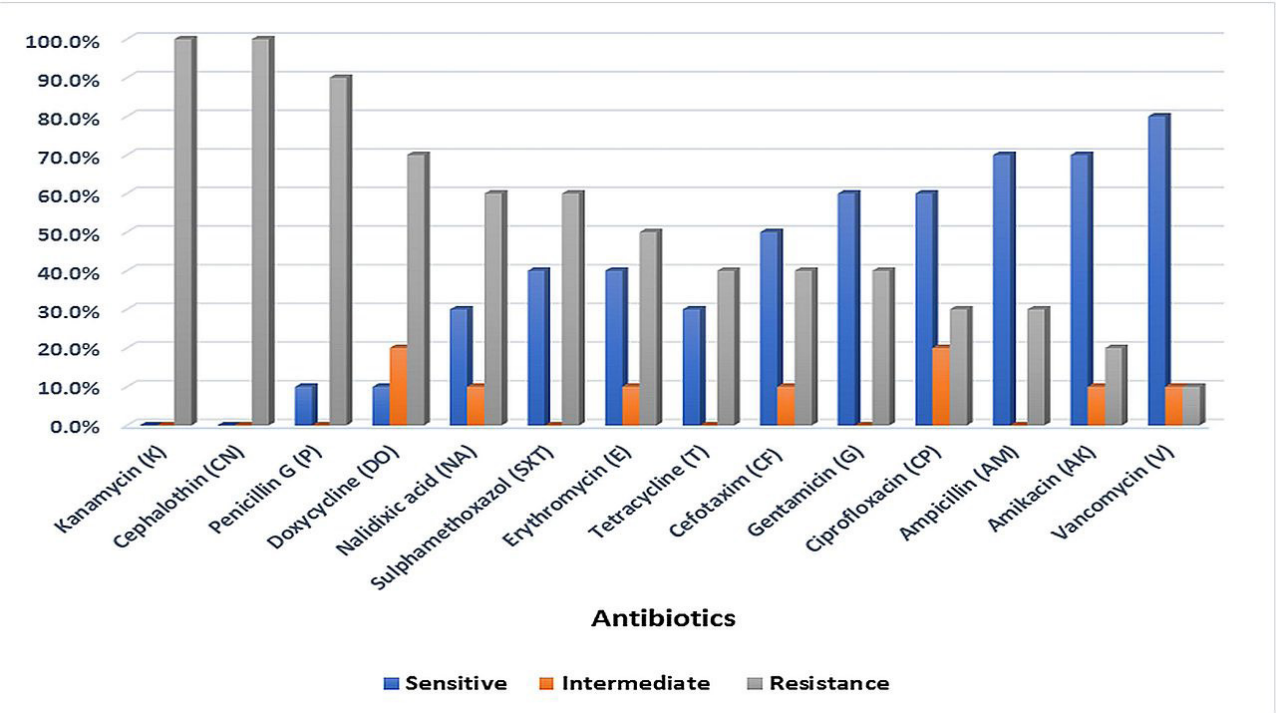
Antibiotic susceptibility of isolated *S. aureus*.

The findings also demonstrated that all isolated *S. aureus* had AMR to at least one antibiotic. AMR to kanamycin and cephalothin was the highest (100%), followed by penicillin (90%), doxycycline (70%), nalidixic acid (60%), sulfamethoxazole (60%), erythromycin (50%), tetracycline (40%), cefotaxime (40%), gentamicin (40%), ciprofloxacin (30%), ampicillin (30%), and amikacin (20%). In comparison, the least resistance was found to vancomycin (10%) (Fig. 4). MAR index ranged from 0.143 to 1 (mean = 0.529) (Tables 10 and 11). This came in agreement with Arslan and Oezdemir [49], who conducted their studies on a total of 135 cheese samples. They demonstrated that *S. aureus* isolates had resistance to ≥1 antimicrobial agent; the greatest AMR was found to ampicillin (41%), penicillin (40.1%), and tetracycline (38.7%). On the other hand, and according to Can et al. [50], all *S. aureus* isolates showed susceptibility to gentamicin, oxacillin, and vancomycin. The greatest resistance was found to penicillin and ampicillin (95% and 92.5%, respectively), followed by tetracycline (30%), erythromycin (20%), and ciprofloxacin (12.5%). 

**Table 10.** Antibiotic resistance of *S. aureus *isolates (*n* = 10).

No.	Strain	Antibiotic resistance	MAR index
**1**	*S. aureus*	Kanamycin, Cephalothin, Erythromycin, Tetracycline, Cefotaxime, Gentamicin, Ciprofloxacin, Ampicillin, Amikacin, Vancomycin.Penicillin, Doxycycline, Nalidixic acid, Sulphamethoxazole	1
**2**	*S. aureus*	Kanamycin, Cephalothin, Erythromycin, Tetracycline, Cefotaxime, Gentamicin, Ciprofloxacin, Ampicillin, Amikacin.Penicillin, Doxycycline, Nalidixic acid, Sulphamethoxazole	0.928
**3**	*S. aureus*	Kanamycin, Cephalothin, Erythromycin, Tetracycline, Cefotaxime, Gentamicin, Ciprofloxacin, Ampicillin Penicillin, Doxycycline, Nalidixic acid, Sulphamethoxazole	0.857
**4**	*S. aureus*	Kanamycin, Cephalothin, Penicillin, Doxycycline, Nalidixic acid, Sulphamethoxazole, Erythromycin, Tetracycline, Cefotaxime, Gentamicin.	0.714
**5**	*S. aureus*	Kanamycin, Cephalothin, Penicillin, Doxycycline, Nalidixic acid, Sulphamethoxazole, Erythromycin.	0.500
**6**	*S. aureus*	Kanamycin, Cephalothin, Penicillin, Doxycycline, Nalidixic acid, Sulphamethoxazole	0.428
**7**	*S. aureus*	Kanamycin, Cephalothin, Penicillin, Doxycycline.	0.286
**8**	*S. aureus*	Kanamycin, Cephalothin, Penicillin.	0.214
**9**	*S. aureus*	Kanamycin, Cephalothin, Penicillin.	0.214
**10**	*S. aureus*	Kanamycin, Cephalothin.	0.143
Average = 0.529			

MDR of *S. aureus* isolates was 90% in this study. By comparison, several other studies reported varying percentages of MDR. For instance, in Turkey, Kayili and Sanlibaba [51] reported MDR in 72.94% of *S. aureus* isolates. Also, MDR was 61.1% in China [52] and 66.67% in USA [53]. 

**Table 11.** MDR of *S. aureus* isolates (*n* = 10).

Item	Number	%
**Isolates with MDR (MAR index > 0.2)**	9	90
**Isolates without MDR (MAR index < 0.2)**	1	10

## Conclusion

The results reveal that Tallaga, Domiati, Cheddar, and Ras cheeses in Egyptian markets show high contamination with *S. aureus* and *E. coli*. The existence of MDR bacteria is worrying since these bacteria may threaten public health. Thus, periodical evaluation of dairy products for ensuring consumer safety should be practiced. Good manufacturing practices and strict personal hygienic measures are mandatory for ensuring the safety and high quality of dairy products. Further studies are essential to be conducted to evaluate whether these strict hygienic measures are applied or not to protect human health, particularly during the current COVID-19 pandemic situation.

## List of Abbreviations

AMR, antimicrobial resistance; DNA, deoxyribonucleic acid; dNTP, deoxynucleoside triphosphate; EHEC, enterohemorrhagic *E. coli*; EIEC, Enteroinvasive *E. coli*; EPEC, Enteropathogenic *E. coli*; ETEC, enterotoxigenic *E. coli*; PCR, polymerase chain reaction; *stx1*, Shiga toxin 1; *stx2*, Shiga toxin 2.
